# Aged induced pluripotent stem cell (iPSCs) as a new cellular model for studying premature aging

**DOI:** 10.18632/aging.101248

**Published:** 2017-05-31

**Authors:** Stefania Petrini, Rossella Borghi, Valentina D'Oria, Fabrizia Restaldi, Sandra Moreno, Antonio Novelli, Enrico Bertini, Claudia Compagnucci

**Affiliations:** 1 Confocal Microscopy Core Facility, Research Laboratories, Bambino Gesu’ Children's Research Hospital, IRCCS, Rome 00146, Italy; 2 Department of Neuroscience, Unit of Neuromuscular and Neurodegenerative Diseases, Laboratory of Molecular Medicine, Bambino Gesu’ Children's Research Hospital, IRCCS, Rome 00146, Italy; 3 Department of Science-LIME, University “Roma Tre”, Rome 00146, Italy; 4 Medical Genetic Unit and Laboratory of Medical Genetics, Bambino Gesu’ Children's Research Hospital, IRCCS, Rome, Italy

**Keywords:** induced pluripotent stem cells, nucleoskeleton, lamins, emerin, nesprins, mitochondria, actin cytoskeleton, MKL1, SIRT7

## Abstract

Nuclear integrity and mechanical stability of the nuclear envelope (NE) are conferred by the nuclear lamina, a meshwork of intermediate filaments composed of A- and B-type lamins, supporting the inner nuclear membrane and playing a pivotal role in chromatin organization and epigenetic regulation. During cell senescence, nuclear alterations also involving NE architecture are widely described. In the present study, we utilized induced pluripotent stem cells (iPSCs) upon prolonged *in vitro* culture as a model to study aging and investigated the organization and expression pattern of NE major constituents. Confocal and four-dimensional imaging combined with molecular analyses, showed that aged iPSCs are characterized by nuclear dysmorphisms, nucleoskeletal components (lamin A/C-prelamin isoforms, lamin B1, emerin, and nesprin-2) imbalance, leading to impaired nucleo-cytoplasmic MKL1 shuttling, actin polymerization defects, mitochondrial dysfunctions, *SIRT7* downregulation and NF-kBp65 hyperactivation. The observed age-related NE features of iPSCs closely resemble those reported for premature aging syndromes (e.g., Hutchinson-Gilford progeria syndrome) and for somatic cell senescence. These findings validate the use of aged iPSCs as a suitable cellular model to study senescence and for investigating therapeutic strategies aimed to treat premature aging.

## INTRODUCTION

Nuclear envelope (NE) is a bilayer membrane enclosing the nucleus contents in eukaryotic cells; it is formed by the inner (INM) and the outer (ONM) nuclear membranes, composed of numerous integral membrane proteins playing pivotal roles in chromatin organization and gene expression. INM proteins include emerin, lamin-associated proteins (LAPs), MAN1, SUN proteins and lamin B receptor (LBR), while ONM contains proteins such as nesprins and KASH domain proteins, involved in nuclear positioning by interactions with actin cytoskeleton [1, 2]. The physical connection between cytoskeleton and nucleus is mediated by the nuclear lamina, a meshwork of intermediate filaments composed of A- and B-type lamins, providing nuclear integrity and mechanical stability, supporting the INM, and playing a crucial role in chromatin arrangement and epigenetic regulation [3, 4]. Lamins expression is regulated during development, in a cell type-specific manner [5]. Particularly, embryonic stem cells do not express A-type lamins and they lack heterochromatin, thus maintaining broad genome plasticity, whereas B-type lamins are ubiquitously expressed [6, 7]. *LMNA* encodes two main variants by alternative splicing, prelamin A and lamin C, sharing the first 566 amino acids [8]. Several human diseases have been linked to mutations affecting *LMNA* or genes encoding B-type lamins, LAPs and other NE proteins. Among these disorders, referred to as “laminopathies”, the most severe phenotype is found in premature aging conditions, such as Hutchinson-Gilford progeria syndrome (HGPS), a rare autosomal dominant syndrome characterized by the appearance of aging hallmarks early in childhood [9]. HGPS is caused by a *LMNA* point mutation responsible for an aberrant prelamin A isoform, called progerin (Δ150lamin A), that tightly associates with the INM, and accumulates intranuclearly, dramatically affecting nuclear architecture and cellular functions. Interestingly, progerin increases gradually during physiological aging and whether this is due to spontaneous *LMNA* mutations, to epigenetic modifications, or to abnormal farnesylation, it is still unclear [10, 11]. Normal aging is a complex biological process characterized by several dysregulated pathways, some of which contributing to premature aging in HGPS, namely mitochondrial and telomere dysfunctions, heterocromatin loss and disorganization, reactive oxygen species (ROS) accumulation and alterations of NE components [12, 13].

Substantial evidence suggests that stem cells dysfunction plays a key role in the pathogenesis of premature aging syndromes and a timely elimination of aged/dysfunctional stem cells is essential to protect individuals from these disorders [14]. Therefore, we decided to unveil the underpinnings of aging in cells considered similar to embryonic stem cells and used as a model for several human pathologies, i.e., induced pluripotent stem cells (iPSCs). These may be obtained from skin fibroblasts, reprogrammed into cells able to self-renew and to differentiate into virtually all cell types, so allowing ‘*in vitro* disease modeling’ of rare genetic disorders and age-related diseases [15–17]. It is accepted that iPSCs can be maintained and propagated indefinitely in culture, maintaining the ability to re-differentiate into fully rejuvenated cells [18, 19]. However, focusing on *in vitro* hallmarks of stem cell aging, we demonstrated that, when cultured for prolonged time (one year), iPSCs display altered mitochondrial number, functionality and biogenesis, and fail to undergo neurogenesis [20]. Highlighting possible biological differences between young (y-) and aged (a-) iPSCs concerning their mitochondrial status and their NE integrity, is certainly relevant to the study of age-related disorders, allowing to dissect the molecular mechanisms underying cell senescence. Furthermore, the use of aged iPSCs may provide important information regarding the influence of *in vitro* environment, in order to design novel therapeutic strategies against premature aging.

## RESULTS

### Increased lamin A/C levels and nuclear dysmorphisms associated with mitochondria are features of aged-iPSCs

In this work we deepened the knowledge of iPSCs biology, studying the behavior of NE constituents both in pluripotent cells (soon after fibroblasts reprogramming, named young-iPSCs or y-iPSCs) and in the same cells cultured for prolonged time (named aged-iPSCs or a-iPSCs), in order to investigate if NE dysfunctions are also induced in pluripotent stem cells by aging. We used iPSCs obtained from fibroblasts of a healthy male adult and, in particular, we used three clones for all the experiments reported in this work. In addition to these, we also used a clone of iPSCs named 19.9 (from the J. Thomson Lab, [21]) before and following aging. To investigate the genetic stability of the lines used, we performed karyotypic analysis and observed a normal karyotype in y-iPSCs (Figure S1A), whereas > 70% of a-iPSCs presented chromosome 1 trisomy (Figure S1A). To confirm the pluripotency status of a-iPSCs, we performed alkaline phosphatase assay (Figure S1B) and immunofluorescence analysis of stemness markers (SSEA4, OCT4, SOX2 and TRA1-60; Figures S1C and S1D) and observed positivity for all stainings on both young and aged iPSCs.

**Figure 1:**
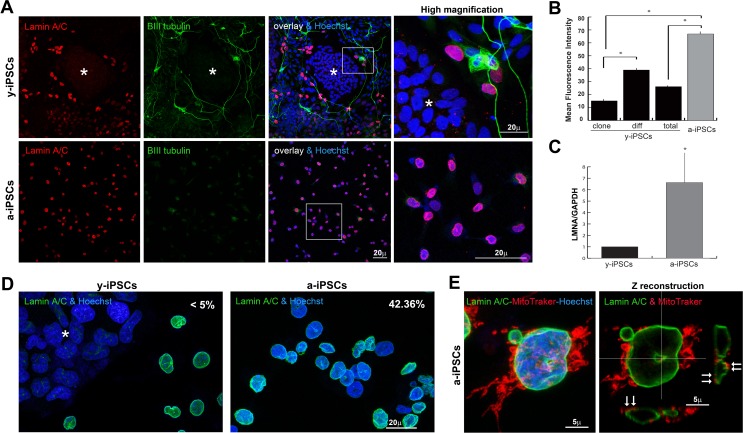
Behaviour of lamin A/C, nucleoskeleton and mitochondria in iPSCs, in pluripotent state and following differentiation and senescence (**A**) Lamin A/C and β-III tubulin staining was absent in y-iPSCs of the colony (*asterisk*), but normally detectable upon differentiation (*red* nuclei). Lamin A/C strongly increased in aged cells, whereas β-III tubulin was faintly present (*right* column: high magnification of the *inset*). (**B**) Mean fluorescence intensity of lamin A/C immunostained nuclei in colony (*clone*) and differentiated (*diff*) cells from y-iPSCs and from a-iPSCs nuclei. Data are presented as the mean ± SEM (*: p < 0,05). (**C**) RT-PCR analysis of LMNA mRNA expression in y-iPSCs and a-iPSCs. Reduced levels of lamin A/C are associated with the youth and stemness (*: p < 0,05, n=3). (**D**) Increased nuclear dysmorphisms were found in a-iPSCs. (**E**) Abnormal accumulation of mitochondria was noticed in association with nucleoskeletal alterations in senescent iPSCs (*arrows*).

In addition to the strategy developed by Miller et al. [22] to induce aging with progerin overexpression, we wanted to identify which NE features, delining the ground-state pluripotency of iPSCs, are involved in premature aging. In line with the literature and the concept that iPSCs have the same biological features of embryonic stem cells, lamin A/C expression lacks in pluripotent cells of the colony and it starts to get organized in the NE of spontaneously differentiated y-iPSCs (Figure 1A). In fact, the β−III tubulin positive cells outside the colony in y-iPSCs dysplay neurons spontaneously differentiated in cultures kept close to confluency. A-iPSCs showed reduced rates of iPS-like colony formation and faintly expressed β−III tubulin, thus confirming their undifferentiated status. A-iPSCs expressed high LMNA mRNA levels, presented a thickened nuclear lamina strongly stained by anti-lamin A/C antibodies (Figures 1A and 1B), as reported in HGPS and senescent cells [13, 23].

Strikingly, altered lamin A/C expression found in a-iPSCs, is associated to nuclear dysmorphisms as doughnut-shaped nuclei, blebs and folded NEs (about 42.36% of total cells, Figure 1D) resembling those of laminopathic cells, as pointed out by 3D rendering of nucleoskeletons of lamin A/C stained cultures using extensive 3D reconstructing software Imaris (Figure S3). Further, mitochondria distribution is mislocated in senescent iPSCs and strictly associated to NE alterations (Figure 1E).

### Prelamin A, progerin and NF-kB are increased, while SIRT7 mRNA levels are reduced, in senescent iPSCs

**Figure 2:**
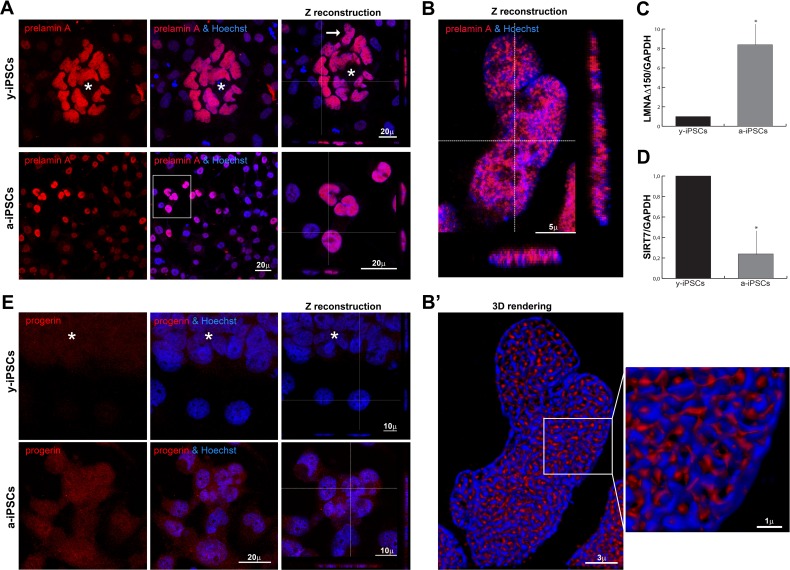
Distribution of prelamin isoforms and expression of LMNAΔ150 and SIRT7 in iPSCs, in pluripotent state and following differentiation and senescence (**A**) Prelamin A was throughly distributed inside iPSCs nuclei of the colony cells (*asterisk*) and aged cells, as represented by XYZ stacks (*right* column), and it colocalized with nucleic acid stain (Hoechst, *blue*), as visualized in high magnifications (of the *inset* for a-iPSCs, and of y-iPSCs nuclei (*arrow*) showed in (**B**). (**B**) XYZ high magnification of y-iPSCs nuclei (indicated in a, *arrow*) showing a clear intranuclear accumulation of prelamin A in pluripotent stem cells. (B’) Deconvolved 3D-rendering of raw Z stack (in **B**) showing tight interconnections between prelamin A and chromatin distribution (high magnification of the inset, *right*). (**C**) Relative expression levels of LMNAΔ150 mRNA expression in y-iPSCs (*: p < 0,05, n=3). (**D**) Relative expression levels of SIRT7 mRNA in y-iPSCs and a-iPSCs (*: p <0,05, n=3). (**E**) Progerin expression was undetectable in y-iPSCs of the colony (*asterisk*) and in differentiated cells, whereas it appeared in senescent iPSCs.

Since prelamin A precursors also accumulate during physiological aging [24–26], we hypothesized that nuclear dysmorphisms in a-iPSCs could be correlated with altered prelamin processing, as described in progeroid cells. To test this hypothesis, we performed full-length prelamin A immunostaining, thereby detecting a brilliant intranuclear labeling in pluripotent cells whereas differentiated cells are negative or weakly stained (Figure 2A). Fine 3D-reconstruction obtained after deconvolution analysis (Figures 2B and 2B’), revealed a close relationship between prelamin A and chromatin distribution.

As expected, its expression highly increased inside a-iPSC nuclei, thus suggesting an imbalanced age-dependent mechanism in the prelamin maturation pathway (Figure 2A). We deepened the prelamin isoforms characterization, detecting a high progerin nuclear accumulation in a-iPSCs as result of senescence (Figure 2E). Accordingly, the expression levels of the progerin transcript (Δ150LMNA) [26], is augmented in a-iPSCs when compared to y-iPSCs. RT-PCR analysis shows that the use of the criptic splice site is about 8-fold higher in a-iPSCs (Figure 2C) suggesting a tight association between nuclear dysmorphisms and increased progerin levels in a-iPSCs, as reported in progeroid cells. These findings corroborate our hypothesis that a-iPSCs present altered biological features, mirroring those of pathological and physiological aging.

Since the increase of inflammatory processes contribute to senescence [27], we investigated the activation status of the transcription nuclear factor-kB (NF-kB). NF-kB is weakly distributed in the cytoplasm of iPSCs belonging to the colony, but it moves to the nuclear compartment upon differentiation; the prolonged *in vitro* culture condition leads to NF-kB hyperactivation in a-iPSCs (Figure S2A). These inflammatory alterations found in a-iPSCs suggested an involvement of sirtuins, known to regulate life span and prevent aging-related diseases, mainly by catalyzing the deacetylation of histones and regulation of many transcription factors, such as NF-kB [28, 29]. In particular, SIRT7 expression, which regulates mitochondrial biogenesis, was found reduced in aged heamatopoietic stem cells [29]. In line with these findings, a-iPSCs have decresead levels of *SIRT7* mRNA (Figure 2D).

### Increased emerin and nesprin-2, and reduced lamin B1 levels characterize aged-iPSCs

**Figure 3:**
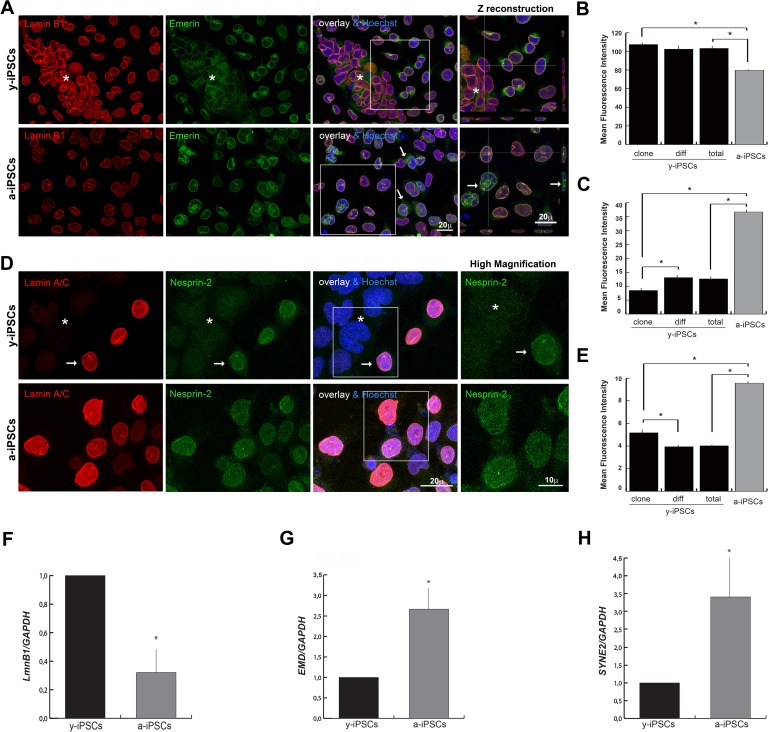
Reduced levels of emerin, and nesprin-2 are associated to the youth and stemness, whereas low expression of lamin B1 is associated to senescence (**A**) Emerin was mildly polymerized and interdispersed around the nuclear rim in colony y-iPSCs (*asterisk*) but normally distributed and expressed in differentiated cells; emerin expression increased upon aging although it keeps mislocalized around nuclei (*arrows*), whereas lamin B1 decreased. Z reconstructions of high magnification of the insets (*right* column). (**B**) Mean fluorescence intensity of emerin immunostained nuclei of colony (*clone*) and differentiated (*diff*) cells from y-iPSCs and from a-iPSCs cultures. Data are presented as the mean ± SEM (*: p < 0,05). (**C**) Mean fluorescence intensity of lamin B1 immunostained nuclei of colony (*clone*) and differentiated (*diff*) cells from y-iPSCs and from a-iPSCs nuclei (*: p < 0,05). (**D**) Lamin A/C and nesprin-2 immunoexpression in colony cells (*asterisk*) and differentiated cells (*arrow*) of y-iPSCs, and in aged cells. High magnification of the insets (*right* column) concerning the nesprin-2 distribution. (**E**) Mean fluorescence intensity of nesprin-2 labeled nuclei of colony (*clone*) and differentiated (*diff*) cells from y-iPSCs and from a-iPSCs cultures (*: p < 0,05) (**F**) Relative expression levels of LMNB1 mRNA in y-iPSCs and a-iPSCs showing higher levels in y-iPSCs compared to senescent cells (*: p < 0,05, n=3). (**G**) RT-PCR analysis of EMD mRNA detected higher levels in senescent cells (*: p < 0,05, n=3). (**H**) Relative expression levels of SYNE2 mRNA in y-iPSCs and a-iPSCs (*: p <0,05, n=3).

Since B-type lamins are present in the nuclear lamina in undifferentiated stem cells [5], we investigated their behaviour performing lamin B1 immunofluorescence and 3D reconstruction analyses. Lamin B1 is constitutively present in y-iPSCs whereas reduced levels have been observed in a-iPSCs (Figures 3A, 3B and 3F, Figure S3), similarly to what reported in HGPS fibroblasts and during cell senescence [31, 32].

Emerin mislocations are reported in lamin A and nesprins mutant cells [33, 34]; in particular, emerin cytoplasmic localization is observed in the ONM and endoplasmic reticulum in *LmnA−/−* and in *Lmna*N195K cells [33]. In line with these observations, we found a high emerin expression in spontaneously differentiated cells distributed around the colony, whereas in pluripotent cells emerin was weakly polymerized and interdispersed around the nuclear rim. In a-iPSCs, emerin polymerization and expression increased although it keeps mislocalized around nuclei (Figures 3A, 3C and 3G; Figure S3).

To finalize the NE analysis, we investigated the levels of nesprins that are actin-binding NE proteins interacting with SUN proteins to form the LINC (linker of nucleoskeleton and cytoskeleton) complex, that is involved in mechanotransduction of intra- and extra-cellular stimuli via the cytoskeleton to the nucleus, in transcriptional regulation, in centrosomal positioning and cell polarization [35]. In particular, nesprin-2 (coded by *SYNE2*) binds the C-terminal common region of lamin A/C and its localization and function at the NE depends on the lamin A/C network. Furthermore, nesprin-2 binds emerin and is crucial for its proper localization [36, 37]. Since nesprin distribution is affected in lamin A/C deficient cells [36], we determined whether nesprin-1/2 expression followed the behavior of lamin A/C. Confocal microscopy showed that nesprin-1 lacks in iPSCs of the colony, in differentiated and a-iPSCs (Figure S2C), whereas nesprin-2 was faintly detected in colony cells, intranuclearly distributed and mildly polymerized at the nuclear rim upon differentiation, but uniformly increased throught the nucleus in a-iPSCs (Figures 3D, 3E and 3H).

Since nesprin-2 may regulate mitochondria distribution [37] and a-iPSCs presented altered gene expression concerning mitochondrial biogenesis [20], we investigated if mitochondria distribution reflects nucleoskeletal alterations. In fact, induced aging in iPSCs causes an abnormal accumulation of mitochondria in close relationship with nuclear dysmorphisms, i.e. in lobulated or doughnut-shaped nuclei (Figures 1E and S2A), thus suggesting a tight association between mitochondria and NE.

### Altered nucleo-cytoplasmic MKL1 shuttling is associated to senescence in iPSCs

It is known that lamin A/C and emerin are implicated in nuclear-cytoskeletal organization through modulation of actin polymerization, thus providing the mechanical stability to the cells [33, 38]. Therefore, we tested if the qualitative (i.e. presence of prelamin isoforms, nuclear dysmorphisms) and quantitative (i.e. expression levels of NE components) alterations of a-iPSCs could be reflected in functional NE alterations, as suggested by experiments performed on laminopathic cells testing the nucleo-cytoplasmic shuttling of the mechanosensitive transcription factor megakaryoblastic leukemia 1 (MKL1), which is localized in the cytoplasm by binding to G-actin. Mitogenic or mechanical stimulation triggers actin polymerization, liberating MKL1 from G-actin and exposing a nuclear localization sequence within MKL1 actin-binding domain. Increased nuclear import, coupled with decreased export, causes MKL1 nuclear accumulation. Since nuclear translocation of endogenous MKL1, in response to serum stimulation, is abrogated in *Lmna−/−* mouse embryonic fibroblasts, the impaired MKL1 translocation is an effect of lamin A/C loss [33]. In line with these prior observations, we found that in y-iPSCs (lacking lamin A/C), the nucleo-cytoplasmic MKL1 shuttling is severely delayed; in fact, after 3 hours of stimulation, MKL1 is still in the cytoplasm, while in a-iPSCs where lamin A/C levels increase, the nucleo-cytoplasm translocation of MKL1 is quicker (Figure S2B). These results suggest a close correlation between lamin A/C expression and functional NE features and, consequently, of G-actin transport in iPSCs.

### Slow actin polymerization rate is associated to senescence in a-iPSCs

**Figure 4:**
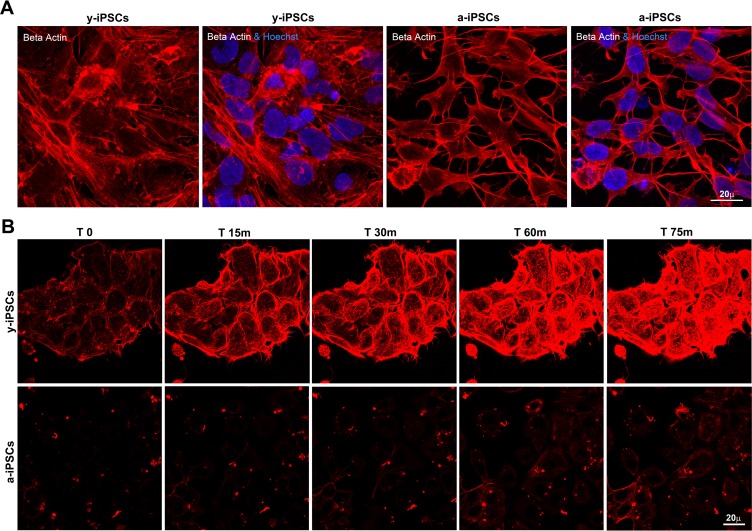
Slow actin polymerization rate is associated to senescence in a-iPSCs (**A**) Basal expression of beta actin in iPSCs, showing defects in cytoskeletal organization of F-actin in aged cells. (**B**) Representative images of time-lapse experiments performed on y-iPSCs and a-iPSCs, after cytochalasin D treatment and loading with SiR-actin probe, to monitor the actin cytoskeleton functionality.

Given the absence of lamin A/C and the low emerin levels at the y-iPSCs NE, we investigated if this condition affects the polymerization process of cytoskeletal filaments. Whereas y-iPSCs showed well-assembled actin filaments, a-iPSCs exibited poor organized and short actin stress fibers (Figure 4A) suggesting age-related defects in nuclear and cytoskeletal stability similarly to those observed in lamin and/or emerin mutant cells. In addition, we deepened the modulation of actin dynamics through the induction of actin re-polymerization after filament disruption with cytochalasin D treatment, performing live-cell imaging experiments. Importantly, XYZt imaging and Imaris-derived 3D rendering of time-lapse experiments revealed different re-polymerization rates of cytoskeleton filaments after treatment, between y- and a-iPSCs (Figure 4B, Movie S1 and Movie S2). In fact, these latter were slower to reassemble actin fibers compared to y-iPSCs. Therefore, induced senescence in iPSCs is reflected in disturbed actin dynamics, similarly to what observed in laminophatic cells. Since low emerin levels seem sufficient to regulate cytoplasmic actin polymerization in iPSCs, our data suggest a primary role for emerin in modulating actin organization and orientation.

## DISCUSSION

Premature aging disorders like Werner syndrome, Bloom's syndrome, and Hutchinson-Gilford Progeria Syndrome (HGPS), are monogenic rare disease characterized by premature aging and reduced lifespan of the children affected. Interestingly, these diseases recapitulate many of the phenotypes observed in physiological aging. Despite the great social and clinical interest, the pathogenetic mechanisms and the identification of efficacious therapeutic remedies are still under investigation [39–41]. Recent works on HGPS have revealed alterations in cellular and molecular pathways involved in the maintenance of the stem cell population and of the genomic integrity, thus suggesting a profound impact of the nuclear lamina in nuclear organization, chromatin dynamics, regulation of gene expression, epigenetics and in the maintenance of the stem cell pool [13].

This work addresses the biology of iPSCs and, in particular, the structural changes occurring during aging. Since we investigated the phenotype of iPSCs after prolonged culture, we considered the possibility that the observed phenotypes could be the consequence of genetic drift. For this reason, we analyzed multiple independently cultured lines derived from the same initial starting population. We performed three independent experiments on each of the three iPSCs clones obtained from the fibroblasts (belonging to a healthy male adult) and on one clone of iPSCs (the 19.9 from the Thomson lab) obtained from a different healthy donor. Human mutations affecting genes encoding for components of the nucleoskeleton or for proteins interacting with it, lead to human premature diseases. Despite our knowledge on the nucleoskeleton has a long history, the molecular details of aged pluripotent stem cells are still lacking. This is mostly due to the impossibility to access this cell type *in vivo*, which would lead to the destruction of a developing embryo. But now this obstacle can be easily overcome with the usage to the induced pluripotent stem cell (iPSCs) technology, which allows to unveil the mechanisms underlying nucleoskeletal organization and modulation before and after differentiation.

In this work we deepened the knowledge of iPSCs biology, studying the behavior of the nuclear envelope constituents both in pluripotent cells (soon after the reprogramming of fibroblasts, named y-iPSCs) and in y-iPSCs kept in culture for prolonged time (named a-iPSCs), in order to investigate if NE dysfunctions are also induced in pluripotent stem cells by aging. Our results show that increased lamin A/C levels and nuclear dysmorphisms associated with altered mitochondria distributions are features of aged-iPSCs and that prelamin A and progerin are increased in senescent iPSCs. Focusing on *in vitro* hallmarks of stem cell age, we previously demonstrated that iPSCs kept in culture for prolonged time present altered mitochondrial number and functionality, altered expression of genes relevant to mitochondria biogenesis, and fail to properly undergo neurogenesis [20]. Moreover, it is known that progerin accumulation may lead to modulation of mTOR and Wnt pathways, with effects in cellular senescence, stem cell turnover, autophagy, protein inflammation, mitochondrial dysfunction and ROS overproduction [42, 43], ultimately contributing to the premature aging phenotype.

In light of the results obtained in this work, together with the evidence reported by [20], we postulate that the oxygen concentration (of 21%) commonly used in the incubators induces cells to adaptative changes that drive them toward senescence. Therefore, further investigations would be necessary to elucidate if prolonged time in culture may lead to adaptation of the iPSCs to the aerobic cell culture environment (which does not mimic the physiological and anaerobic stem cell niche), and consequently to nucleoskeletal alterations and mitochondrial abnormalities.

In addition to the augmented prelamin A and progerin levels, we observe increased emerin and nesprin-2 expression, and reduced lamin B1 in aged-iPSCs. Importantly, progerin expression has been documented to decrease the expression levels of lamin B1 [13].

Our findings provide evidence that low emerin and nesprin-2 expression, and lack of mature lamin A/C are salient NE hallmarks outlining the ground-state pluripotency of iPSCs. Strikingly, the prelamin precursor accumulation appears a salient hallmark of pluripotent cell nuclei. Conversely, induction of differentiation is accomplished by maturation and proper localization of nuclear lamin A/C at the NE, together with normal polymerization of emerin and nesprin-2. Since low emerin levels seem sufficient to regulate cytoplasmic actin polymerization in pluripotent stem cells, our data suggest a pivotal role for emerin to modulate the organization and orientation of actin flow. In addition to these features, the senescent iPSCs present reduced mRNA levels of SIRT7, NF-kBp65 hyperactivation and altered nucleo-cytoplasmic MKL1 shuttling, associated with a slow actin polymerization rate that accounts for a decreased dynamism of the cytoskeleton. The observed NF-kBp65 hyperactivation and SIRT7 downregulation reinforce the involvement of sirtuins, molecules known to regulate life span and prevent aging-related diseases (mainly by catalyzing the deacetylation of histones and regulation of many transcription factors such as NF-kB) in aging [28, 29].

**Figure 5:**
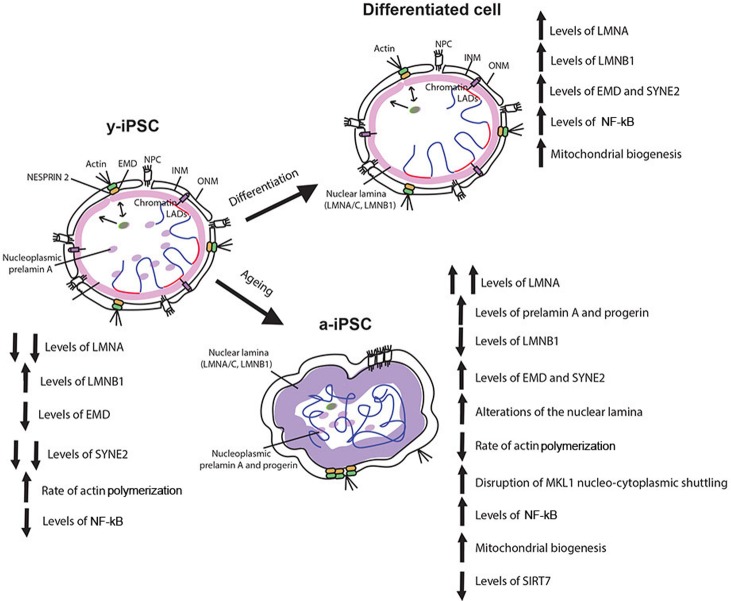
Schematic drawing of the age-associated changes occurring in iPSCs following senescence Behaviour of nuclear envelope components, mitochondria, actin cytoskeleton and MKL1, NF-kB and SIRT7 in y-iPSCs and their changes following differentiation (differentiated cells) and aging (a-iPSCs). Lamin A appears following cell differentiation whereas its overexpression characterized cell senescence. Augmented levels of prelamin A and progerin are present and widely distributed in nuclear matrix of senescent cells. Lamin B1 is normally polymerized in the nuclear lamina of y-iPSCs and differentiated cells, but reduced in aged cells. Emerin is mildly polymerized and interdispersed around the nuclear rim in colony y-iPSCs, normally distributed upon differentiation, but increased in aged iPSCs. Whereas nesprin-1 is not expressed in iPSCs, nesprin-2 appears following differentiation and increases upon cell aging. Mitochondria are abnormally accumulated in senescent iPSCs, and their distribution seems associated to nucleoskeletal alterations. SIRT7 expression, regulating mitochondrial biogenesis, is reduced in aged iPSCs. Altered nucleo-cytoplasmic MKL1 shuttling, associated with a slow actin polymerization rate that accounts for a decreased dynamism of the cytoskeleton are observed in senescent cells. As inflammatory processes contribute to senescence, NF-kB is hyperactivated by prolonged *in vitro* culturing (at 21% oxygen).

Our data demonstrate that upon prolonged *in vitro* culture, iPSCs accumulate a series of age-associated changes mimicking those characterizing pathological and physiological aging (Figure 5) [12, 13], pointing to their utility as a cellular model to study these processes and to develop new therapeutic strategies, thus paving the way for future clinical applications. We observed that aged-iPSCs present alteration of the normal karyotype with chromosome 1 trisomy, and this alteration may be a consequence of the altered expression of NE components observed in these cells. In fact, it is known that progerin accumulation results in disruption of functions of some replication repair factors, causing DNA damage accumulation [44] and that genomic instability and altered chromatin organization are known features of aging cells [12, 13]. Additionally, in line with the observed chromosome 1 trisomy, aged mouse brains present chromosomal aneuploidy [45]. Moreover, *LMNA* gene is located at position 1q22, therefore chromosome 1 trisomy may account for an overexpression of *LMNA* thus leading to high levels of lamin A protein, and importantly, it has been demonstrated that excessive accumulation of lamin A induces mild HGPS-like defects [11]. Therefore, the question, whether the genomic alteration observed in a-iPSCs is a consequene or a cause of the altered expression of the NE components, remains open.

Investigating the features of premature aging in iPSCs is compulsory as these are the closest existing cells to those possibly responsible for the disease, the stem cells. Interestingly, progeroid syndromes may also reflect alterations of the same pathways/properties occurring during physiological aging. In fact, the potential of the progerin is so broad that it was used to induce age-related features to iPSCs, using this characteristic to study the neurodegenerative and age-related disorder Parkinson's disease [22]. Given that reprogramming somatic cells resets the cells back to their embryonic age, modeling age-related disorders with iPSCs technology is a challenge. In addition to the strategy developed by Miller at al [22]. to induce aging with progerin overexpression in human iPSC-derived lineages, we obtained aging in pluripotent stem cell by long term culturing. This cellular model represents an additional resource to study aging in HGPS-derived pluripotent cells, where progerin-induced senescence could not be accomplished as it would alter the intrinsic biology of the HGPS cells. For example, we hypothesize that the aged phenotype of HGPS-iPSCs will appear sooner than in control iPSCs. The methodology reported in this study does not imply transfection or genome editing, but the “induced aging” is achieved by leaving the cells in the environment of common cell culture incubators (5% CO_2_, 21% O_2_) without any manipulations, mimicking physiological and pathological aging in regard of the expression of markers associated to senescence. These results are likely obtained as an adaptation of the iPSCs to the aerobic environment of the incubators, thus suggesting that to avoid induced aging it may be necessary to grow iPSCs in hypoxic environment (closer to the stem cells physiological niche). We believe that further studies are necessary to unveil the biology of iPSCs before their consideration for therapeutic aims.

Here, we propose and characterize a new cellular model for studying aging processes and for modelling late-onset pathologies (i.e. neurodegenerative disorders), represented by iPSCs maintained in culture for prolonged time (at 21% oxygen). This long term culturing strategy may be an attractive solution to investigate biological processes involved in aging, and it stimulates its use in combination with iPSCs derived from patients with HGPS or other degenerative diseases to verify the therapeutic efficacy of drugs known for their promising effects in reverting age-related phenotypes [15, 40]. Therefore, using aged-iPSCs (kept in aerobic condition) for pharmacological treatments may reveal their efficacy as anti-aging drugs. In addition, culturing iPSCs obtained from patients with premature aging syndromes at 21% oxygen may reveal a senescent phenotype that so far has not been studied, as they manifest aging features only following differentiation [14]. This would allow to study the molecular and cellular properties of patients’ aged-pluripotent cells, which are considered responsible for lack of regeneration in specific tissues (for stem cell pool exhaustion) [46].

In conclusion, aged-iPSCs constitute a useful tool for understanding the molecular basis of premature and late-onset age-related pathologies and may constitute a valid model to recapitulate the biology of aging.

## MATERIALS AND METHODS

### Cell lines and culture

Human iPSC lines are purchased from System Biosciences, obtained from skin fibroblasts of a healthy male adult and reprogrammed using non-integrating episomal technology (CS990iPS-1, Minicircle DNA and mc-iPS Cells, Euroclone). iPSCs are generated using the minicircle DNA technology (Cod SC301A-1, circular non-viral DNA), containing cDNAs of human NANOG, SOX2, OCT4, LIN28 genes in vector as described [47]. Three clones of iPSCs were obtained from fibroblasts and were used for all the experiments reported in this work in at least three independent replicates. We considered young (y-iPSCs) those lines cultured for up to 10 passages, while aged (a-iPSCs) lines were derived from y-iPSCs cultured for more than one year in 5% CO_2_, 21% O_2_, at 37°C as described by [20]. Following thawing, iPSCs are grown on MEFs (Life Technologies, Carlsbad, CA) for two passages and then in feeder free condition using Matrigel (BD Biosciences) in mTeSR1 (Stemcell Technologies). We also used the healthy male iPSC line named 19.9 (from the J. Thomson Lab [21],) at early passages and after 50 passages (observing the same phenotype observed in CS990iPS-1 clones). When the iPSCs are 70-80% confluent, they are passaged 1:4 and transferred to new wells in feeder-free condition and incubated at 37°C, 5% CO_2_, the medium is changed every day and the cells split every three days.

### Immunofluorescence analysis

Cultured cells grown on coverslips are fixed in 2% paraformaldehyde in PBS for 10 min followed by cold methanol (-20°C) for 5 min. The following primary antibodies and conditions are used: mouse anti-lamin A/C (used to 1:10 dilution, overnight, sc-7292), goat anti-lamin A/C (1:15, overnight, sc-6215), goat anti-prelamin A (1:100, overnight, sc-6214) purchased from Santa Cruz Biotechnology (Temecula, CA, USA), mouse anti-progerin (1:10, overnight, clone 13A4, ALX-804-662, Enzo Life Sciences, CH), mouse anti-emerin (1:30, 2h, Leica, Mannheim, Germany), rabbit anti-lamin B1 (1:500, overnight, ab16048, Abcam, Cambridge, UK), mouse anti-nesprins 1 and 2 (1:50, overnight, MANNES1A-clone 7A12 and MANNES2G-clone 4B5 are a generous gift of Dr. Lam Le of the Wolfson Centre for Inherited Neuromuscular Disease), monoclonal rabbit anti-NF-kBp65 (1:400, overnight, #8242, Cell Signaling Technology, Danvers, MA), polyclonal rabbit anti-Beta actin (1:200, 2h, ADI-CSA-400, Enzo Life Sciences), anti-β III tubulin (1:500, 2h, #5568, Cell Signaling Technology) and anti-MKL1 (1.100, overnight, HPA030782, Sigma-Aldrich, St. Louis, MO) antibodies. Secondary antibodies conjugated with Alexa Fluor-488, -555 and -647 dyes (Life technologies) are used diluted in 1% PBS/BSA for 1h, RT. Mitochondria and nuclei are stained using Mitotraker (M7512) and Hoechst 33342 (Life technologies) respectively, according to manufacturer instructions. Immunofluorescences with antibodies against the stemness markers OCT4, SSEA4, SOX2, TRA-1-60 are performed using the manufacturer guidelines (PSC 4-Marker Immunocytochemistry Kit, Cat. No no. A24881, Life technologies). Slides are mounted with PBS/glycerol 1:1. Negative controls are performed in each labeling using 1% PBS/BSA without the primary antibody, to verify specific staining. At least three independently iPSC lines obtained from healthy individuals after one and 12 months in culture (in 5% CO_2_ and 21% O_2_) are examined.

### F-actin assay

Cells are treated with 100nM Cytochalasin D (C2618, Sigma Aldrich) for 1 h at 37°C in complete culture medium; after three washes of medium, SiR-actin probe (SC001, Spirochrome, Cytoskeleton, Denver, CO) is added to the medium at final concentration of 1 μM and live cell imaging is performed on a Leica TCS-SP8X laser confocal microscope as described below.

### Confocal microscopy, time-lapse microscopy and image analysis

Confocal microscopy is performed on a Leica TCS-SP8X laser-scanning confocal microscope (Leica Microsystems, Mannheim, Germany) equipped with tunable white light laser (WLL) source, 405nm diode laser, 3 Internal Spectral Detector Channels (PMT) and 2 Internal Spectral Detector Channels (HyD) GaAsP. Sequential confocal images are acquired using a HCPLAPO 63x oil-immersion objective (1.40 numerical aperture, NA, Leica Microsystems) with a 1024×1024 format, scan speed 400Hz, and z-step size of 0.25 μm. Z-reconstructions were imported into Imaris (version 8.2, Bitplane AG, Switzerland) software to obtain their 3D surface rendering using Surpass mode. To improve contrast and resolution of confocal raw images, deconvolution analysis (3D Deconvolution software, Leica Microsystems) is applied to Z stacks before 3D reconstruction. Lasers’ power, beam splitters, filter settings, pinhole diameters and scan mode are the same for all examined samples of each staining. Confocal time-lapse microscopy is performed using the 633 nm laser line of tunable WLL and acquired with a HCPLAPO 63x oil immersion objective (1.40 NA, Leica Microsystems). Z-reconstructions of serial single optical sections are acquired every 2.5 min, and carried out with a 512×512 format, scan speed 400Hz, a confocal scanning zoom magnification up to 1.25, and z-step size of 0.25 μm. During live cell imaging, cells are mantained in a stage incubator (OkoLab, Naples, Italy) at stable conditions of temperature, CO2 and humidity.

The fluorescence intensity average was calculated using the MetaMorph software (Molecular Devices, Inc.) in at least three digital images acquired under 20x magnification, randomly selected and analyzed for each immunostaining. Over 140 cells were counted for each sample analyzed. Tables of images are processed using Adobe Photoshop CS4 software (Adobe Systems Inc).

### Karyotyping

Cells were cultures to exponential growth rate, before harversting IPS were incubated at 37°C with colcemid (Sigma Aldrich) at 1ug/ml (final concentration) for 1,5- 3h. Then the cells were fixed and spread according to standard procedure. Chromosome analysis was performed on human iPSCs by conventional G-banded techniques (300-400 band resolution). The slides were analyzed with Eclipse 80i (Nikon Instruments Europe B.V.) and images were captured using the software Genikon (Nikon Instruments S.p.a.). Results were described in accordance with the ISCN 2016.

### Alkaline phosphatase activity assay

Cultured cells grown on coverslips are fixed in 4% paraformaldehyde in PBS for 10 min. The phosphatase alkaline test is performed using guidelines of Leukocyte Alkaline Phosphatase Kit based on naphthol AS-BI and fast red violet LB (86R-1KT, Sigma). The cells were photographed using a Leica DM1000 equipped with Leica LAS X software.

### RNA isolation, reverse transcription (RT-PCR) analysis

Total RNA is extracted from iPSCs with the single-step acid phenol method using TRIzol (Invitrogen, Carlsbad, CA pn: 15596018) according to the manufacturer's instructions. Each RNA sample is DNase treated (Recombinant DNase I, AM2235 – Ambion) and quantified by NanoDrop 2000 (Thermo Scientific). The reverse transcription reaction is performed in 20 µl starting from 1 µg of total RNA and cDNA was generated by ImProm-II Reverse Transcription System (A3800 – Promega, Madison, WI, USA) or Superscript II reverse transcriptase (18064, Life Technologies) using random hexamers. Three independent RT-PCRs (reverse transcriptase-polymerase chain reactions) are performed for each sample.

### Quantitative real-time polymerase chain reaction

Gene specific exon-exon boundary PCR products (TaqMan gene expression assays, Applied Biosystems) are measured by means of a PE Applied Biosystems PRISM 7700 sequence detection system during 40 cycles. GAPDH mRNA is used for normalization and relative quantification of gene expression is performed according to the ∆∆Ct method. Expression levels are represented in arbitrary units calculated as a relative-fold increase compared to the control sample arbitrarily set to 1. Quantitative RT-PCRs are repeated in triplicates from at least three independent experiments. The primers used are reported in Table 1.

**Table 1:** Primers used in quantitative RT-PCRs independent experiments

	Forward	Reverse
***GAPDH***	5′ – GATGACATCAAGAAGGTGGTG – 3′	5′ – GTCATACCAGGAAATGAGCTTG – 3′
***SYNE2***	5′ - CTCTTCCAGAGCTTCACGAGG – 3′	5′ - CCATCTGCACCAGCCAGGCAC – 3′
***EMD***	5′- GAGTGCAAGGATAGGGAACG – 3′	5′ - GAGGTGGAGGAGGAAGTAGA – 3′
***LMNB1***	5′ – AAGCAGCTGGAGTGGTTGTT – 3′	5′ - TTGGATGCTCTTGGGGTTC – 3′
***LMNA/C***	5′ – GGTGGTGACGATCTGGGCT – 3′	5′ – CCAGTGGAGTTGATGAGAGC – 3′
***Δ150LMNA***	5′ – GCGTCAGGAGCCCTGAGC – 3′	5′ – GACGCAGGAAGCCTCCAC – 3′
***SIRT7***	5′ – CGCCAAATACTTGGTCGTCT – 3′	5′ - CCCTTTCTGAAGCAGTGTCC – 3′

### Statistical analysis

Results are referred from at least three independent experiments on each iPSC line obtained from healthy individuals after one and 12 months in culture (in 5% CO2, 21% O2). Data are expressed as mean and standard deviation. Comparisons between groups are performed by two-tailed unpaired student's t-test and p values <0.05 were considered statistically significant. Data are analyzed using Windows XP Excel.

## SUPPLEMENTARY MATERIAL






