# Supplementary material for: Aged induced pluripotent stem cell (iPSCs) as a new cellular model for studying premature aging
Source: Aging (Albany NY). 2017 May 31;9(5):1453–66. doi: 10.18632/aging.101248 (PMC5472744; doi:10.18632/aging.101248)
Supplement: Supplementary file 1 [file aging-09-1453-s001.pdf]

SUPPLEMENTARY MATERIAL

FIGURES

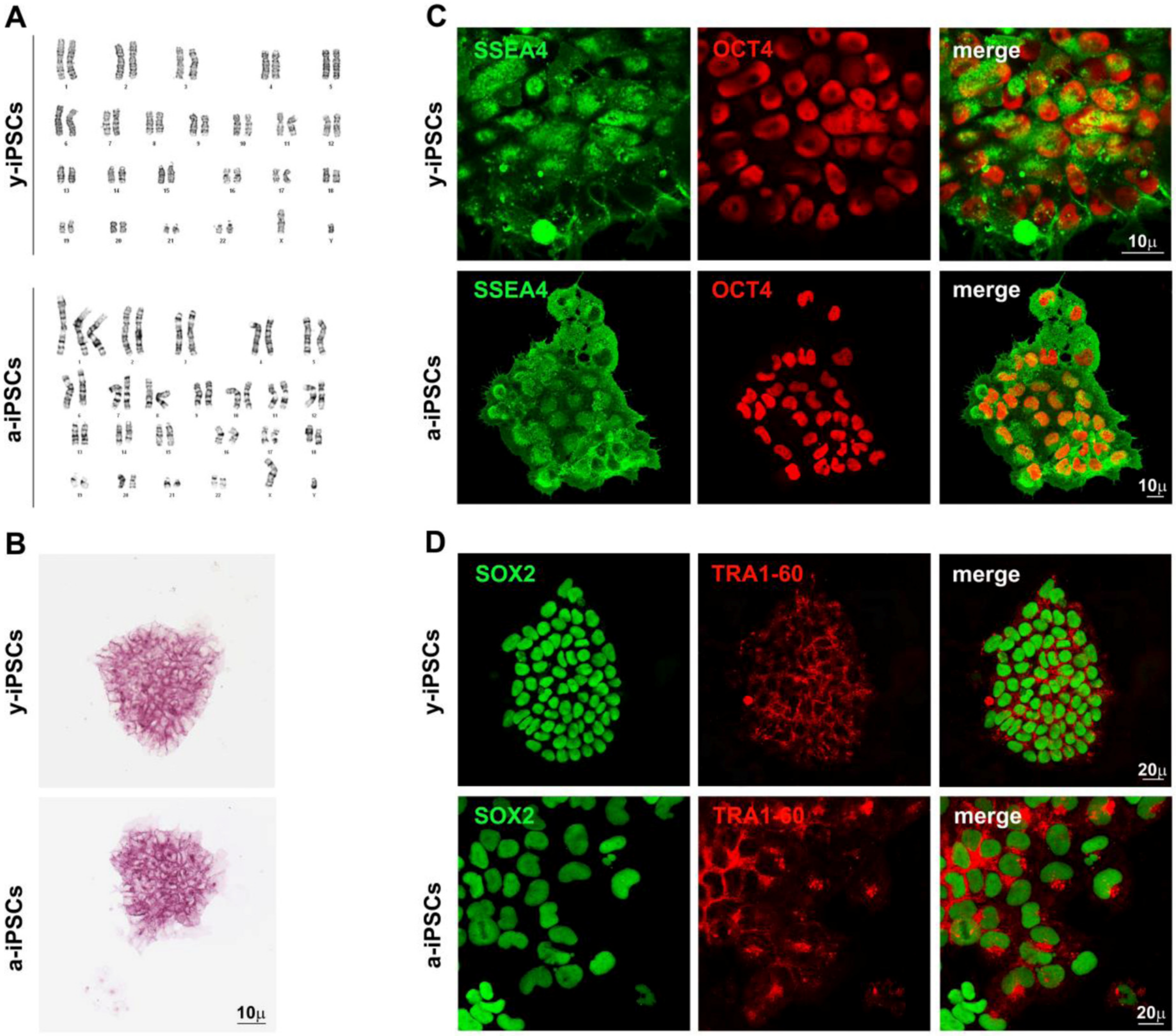

Supplementary Figure S1. Shows karyotype and pluripotency status of young and aged iPSCs. (**A**) Karyogram of y-iPSCs (46,XY - normal male) with normal 46 chromosome human karyotype. a-iPSCs (47,XY,+1 - a karyotype with trisomy 1) karyotype is a 47 chromosome human karyotype with trisomy of chromosome 1. (**B**) Alkaline phosphatase assay of y-iPSCs and a-iPSCs shows positivity on both samples. (**C**) Immunofluorescence analyses of young and aged iPSCs with antibodies against SSEA4 (green) and OCT4 (red) show a similar labelling in both samples. (**D**) Immunofluorescence analysis with antibodies against stemness markers revealed nuclear (SOX2, green) and cytoplasmic (TRA1-60, red) reactions in both young and aged iPSCs.

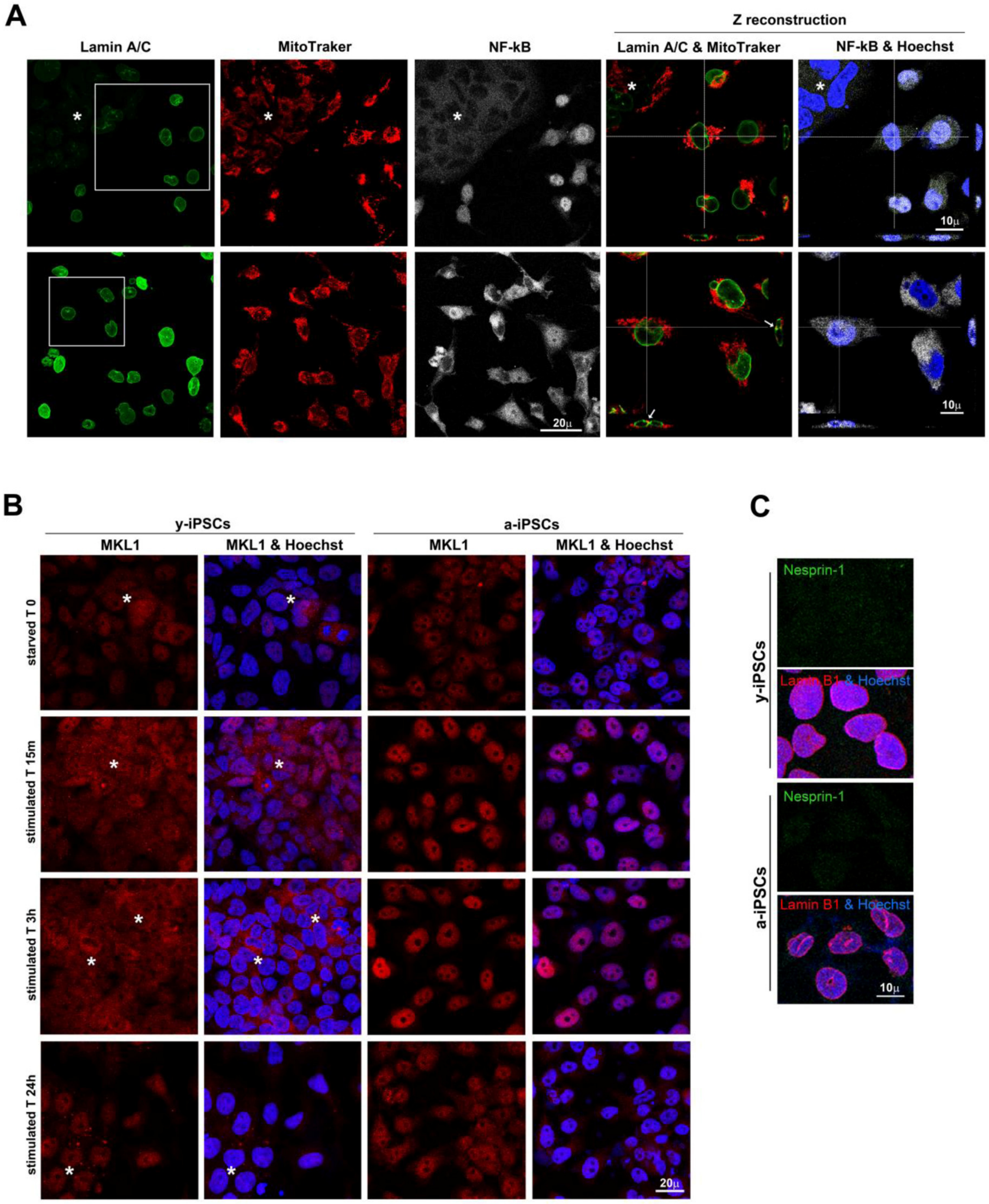

Supplementary Figure S2. Shows that abnormal mitochondria accumulation and distribution, NF-kB hyperactivation and altered nucleo-cytoplasmic MKL1 shuttling are associated to senescence. (**A**) Lamin A/C, mitochondria and NF-kB distributions in colony (asterisk) and differentiated cells of y-iPSCs (upper panel), and in a-iPSCs (lower panel). Abnormal mitochondrial accumulation was observed in association with nuclear lamina dysmorphisms in senescent cells (arrows), in which NF-kB was highly activated. Z reconstructions of high magnification of the insets (right columns). (**B**) Nuclear MKL1 translocation in response to serum stimulation in y-iPSCs of the colony (asterisk) and in neighboring cell was delayed compared to senescent iPSCs.

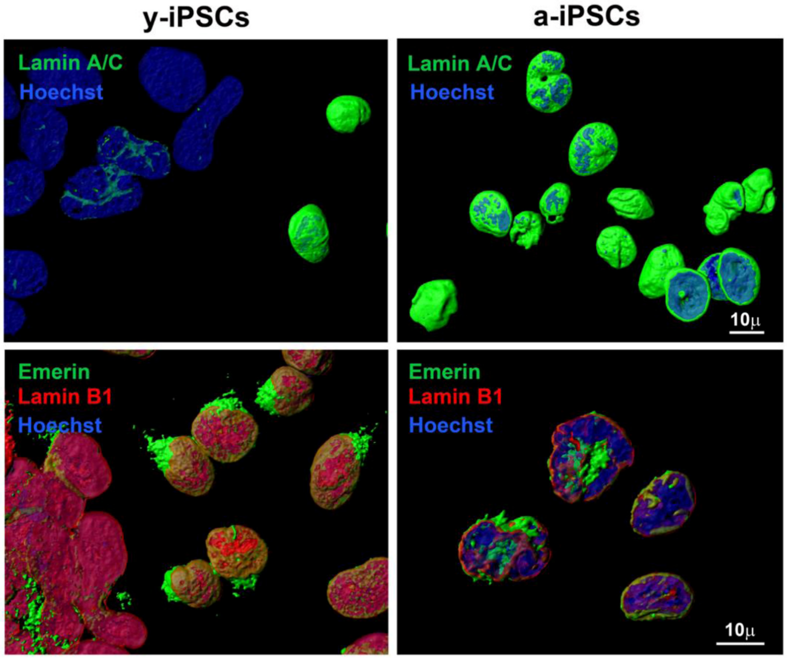

Supplementary Figure S3. Shows the 3D rendering obtained using extensive three-dimensional reconstructing software IMARIS, of young and aged iPSCs immunostained with antibodies against lamin A/C (as shown in Figure 1C), emerin and lamin B1 (reported in Figure 3A).

VIDEOS

Please browse the Full text version to see the video related to this manuscript:

Video S1 shows the three-dimensional rendering performed using Imaris software, of confocal live cell imaging of y-iPSCs, from the same XYZt series that appears in Figure 4B. Polymerization rate of actin cytoskeleton filaments of y-iPSCs, after cytochalasin D treatment, detected by SiR-actin probe.

Video S2 shows the Imaris-derived 3D rendering of confocal live cell imaging of a-iPSCs, from the same time-lapse experiment showed in Figure 4B. Polymerization rate of actin cytoskeleton filaments of y-iPSCs, after cytochalasin D treatment, detected by SiR-actin probe.
